# Modeling Nonlinear Conditional Dependence Between Response Time and Accuracy

**DOI:** 10.3389/fpsyg.2018.01525

**Published:** 2018-09-07

**Authors:** Maria Bolsinova, Dylan Molenaar

**Affiliations:** ^1^ACTNext, ACT, Inc., Iowa City, IA, United States; ^2^University of Amsterdam, Amsterdam, Netherlands

**Keywords:** response time, conditional dependence, nonlinear relationship, response processes, joint modeling, hierarchical model, response moderation

## Abstract

The most common process variable available for analysis due to tests presented in a computerized form is response time. Psychometric models have been developed for joint modeling of response accuracy and response time in which response time is an additional source of information about ability and about the underlying response processes. While traditional models assume conditional independence between response time and accuracy given ability and speed latent variables (van der Linden, 2007), recently multiple studies (De Boeck and Partchev, 2012; Meng et al., 2015; Bolsinova et al., 2017a,b) have shown that violations of conditional independence are not rare and that there is more to learn from the conditional dependence between response time and accuracy. When it comes to conditional dependence between time and accuracy, authors typically focus on positive conditional dependence (i.e., relatively slow responses are more often correct) and negative conditional dependence (i.e., relatively fast responses are more often correct), which implies monotone conditional dependence. Moreover, most existing models specify the relationship to be linear. However, this assumption of monotone and linear conditional dependence does not necessarily hold in practice, and assuming linearity might distort the conclusions about the relationship between time and accuracy. In this paper we develop methods for exploring nonlinear conditional dependence between response time and accuracy. Three different approaches are proposed: (1) A joint model for quadratic conditional dependence is developed as an extension of the response moderation models for time and accuracy (Bolsinova et al., 2017b); (2) A joint model for multiple-category conditional dependence is developed as an extension of the fast-slow model of Partchev and De Boeck (2012); (3) An indicator-level nonparametric moderation method (Bolsinova and Molenaar, in press) is used with residual log-response time as a predictor for the item intercept and item slope. Furthermore, we propose using nonparametric moderation to evaluate the viability of the assumption of linearity of conditional dependence by performing posterior predictive checks for the linear conditional dependence model. The developed methods are illustrated using data from an educational test in which, for the majority of the items, conditional dependence is shown to be nonlinear.

## Introduction

When psychological and educational tests are presented in a computerized form, it is feasible to not only record the product of the response process (i.e., response accuracy), but also the characteristics of the process itself. The most commonly used process variable is response time. Various psychometric models have been developed to jointly model response accuracy and response time (van der Linden, 2007; Molenaar et al., 2015a,b) which are aimed at (1) better measurement of the ability of interest, and (2) investigating the relationship between the time and accuracy components of the process. The prominent framework for modeling the joint distribution of response time and accuracy is the hierarchical modeling framework (van der Linden, 2007), which specifies separate measurement models for ability and speed and combines them on the higher level through the dependence between speed and ability. In this way, when it comes to the relationship between time and accuracy on the same item, the only thing that the model estimates is the correlation between the latent variables—speed and ability—and all the observed correlations between time and accuracy are assumed to be explained by that correlation. That is, conditional on speed and ability, time and accuracy are assumed to be independent. However, it has been shown in multiple empirical data sets (Meng et al., 2015; Bolsinova and Maris, 2016; Bolsinova and Tijmstra, 2016; Bolsinova et al., 2017a,b) that time and accuracy are in fact not conditionally independent and there is more to learn from the conditional dependence between response time and accuracy.

Several methods have been proposed for testing the assumption of conditional independence (van der Linden and Glas, 2010; Bolsinova and Maris, 2016; Bolsinova and Tijmstra, 2016) and different extensions of the hierarchical model have been proposed to relax this assumption (Ranger and Ortner, 2012; Meng et al., 2015; Bolsinova et al., 2017a,b). From these studies, it appears that a violation of conditional independence is not a rare finding and that substantively interesting phenomena may be revealed by investigating the conditional dependencies (Bolsinova et al., 2017c).

When it comes to conditional dependence between time and accuracy, authors typically focus on positive conditional dependence (i.e., relatively slow responses are more often correct) and negative conditional dependence (i.e., relatively fast responses are more often correct). This implies, that a monotone conditional dependence is assumed for time and accuracy. Moreover, most existing models specify the relationship to be linear. However, this assumption of monotone and linear conditional dependence does not necessarily hold in all situations. It could be that responses which are faster than expected are less often correct than responses with response times close to what is expected, but responses slower than expected are not more often correct than those with response times close to what is expected. Therefore, researchers should be able to test whether linearity of conditional dependence between time and accuracy is plausible and to investigate potential nonlinear conditional dependence.

Nonlinear conditional dependence is interesting from the substantive point of view because by abandoning the assumption of monotonicity and linearity of the conditional relationship between time and accuracy one can get a more complete picture of the response process. Since a linear model can only reveal positive or negative dependence, it may ignore important parts of the response phenomena. Imagine a situation in which an item is solved either using a fast optimal strategy or a slow error-prone strategy (i.e., slow responses are less often correct than relatively fast responses) and, in addition to that, some of the respondents respond to the item by guessing (i.e., very fast responses are rarely correct). If one of these phenomena is much stronger than the other, then a linear effect in one of the directions would be detected (i.e., positive conditional dependence if guessing is the strongest factor, or negative conditional dependence if the difference in strategies is the strongest factor). The linear model might also find no evidence of conditional dependence if the two opposing factors balance each other out. In none of these scenarios, a valid conclusion about the relationship between time and accuracy would be drawn. On the contrary, nonlinear methods would allow one to detect a violation of conditional dependence and to get a better understanding of the response processes.

In this paper we develop methods for exploring nonlinear conditional dependence between response time and accuracy. Three different approaches are proposed. (1) The joint models for conditional dependence between time and accuracy (see e.g., Bolsinova et al., 2017b) are extended to include quadratic effects, which allows one to study nonlinear relationships between residual time and accuracy. (2) Partchev and De Boeck's (2012) model is extended to allow for multiple categories of responses which makes it possible to reveal nonmonotonic relationships between time and accuracy. Moreover, the model is modified in such a way that response time is treated as a continuous variable following a log-normal distribution, and the categories are defined based on the difference between the observed and expected log-transformed response time. This allows one to study the conditional dependence separately from the relationship between speed and ability on the higher-level of the hierarchical model. Bayesian estimation algorithms are developed for the two new joint models for response time and accuracy. (3) We propose using the indicator-level nonparametric moderation method (Bolsinova and Molenaar, in press) with residual log-response time as a predictor for the intercept and the slope of the item characteristic curve (ICC), such that nonparametric relationships between the residual response time and the item parameters can be investigated. Furthermore, we propose using nonparametric moderation to evaluate the viability of the assumption of linearity of conditional dependence. This can be done by performing posterior predictive checks for the linear conditional dependence model.

The remainder of the paper is organized as follows. In section 2 the hierarchical model for response time and accuracy is presented and the assumption of conditional independence is formally defined. In section 3 existing models for conditional dependence are discussed. In section 4 we propose three methods for exploring nonlinear conditional dependence. Section 5 presents an empirical example in which nonlinear conditional dependence is investigated, and the paper concludes with a discussion.

## Jointly modeling response time and accuracy using the hierarchical model

In the hierarchical model (van der Linden, 2007; Van Der Linden, 2009) the random variables response accuracy and response time of person *p* on item *i*, denoted by *X*_*pi*_ (with realizations *x*_*pi*_ = 0/1 for incorrect/correct) and *T*_*pi*_ (with realizations *t*_*pi*_), respectively, are assumed to be independent, conditional on the latent variable ability, denoted by θ_*p*_, and speed, denoted by τ_*p*_:

(1) $$\begin{array}{r} f\left(x_{pi},t_{pi}|\theta_{p},\tau_{p}\right)=f\left(x_{pi}|\theta_{p},\tau_{p}\right)f\left(t_{pi}|\theta_{p},\tau_{p}\right). \end{array}$$

Furthermore, it is assumed that response accuracy is independent of speed given ability, and that response time is independent of ability given speed. The full specification of the hierarchical model for response times and accuracy requires four model ingredients: (1) a measurement model for response accuracy, typically an item response theory (IRT) model; (2) a measurement model for response times; (3) a model for the relationship between the latent variables; and (4) a model for the relationship between the item parameters. In this section, we will present a simple specification of the model, which we will use as a basis for describing the existing extensions of the hierarchical model allowing for conditional dependence.

For the response accuracy measurement model, we use a two-parameter normal-ogive model (Lord and Novick, 1968) in which the probability of a correct response to the item depends on the ability of the person:

(2) $$\begin{array}{r} \mathrm{Pr}\left(X_{pi}=1|\theta_{p}\right)=\text{Φ}\left(\alpha_{i}\theta_{p}+\beta_{i}\right), \end{array}$$

where *α*_*i*_ and *β*_*i*_ are the slope and the intercept of the ICC, and Φ(·) denotes the cumulative standard normal distribution function. Alternatively, the three-parameter normal-ogive model (Klein Entink et al., 2009), logistic IRT models (Bolsinova et al., 2017a), and cognitive diagnostic models (Zhan et al., 2018) have been used as the first ingredient for the hierarchical model.

For the response time measurement model, we use a log-normal model (van der Linden, 2006) in which the response times are assumed to have a log-normal distribution with the mean equal to the difference between the time intensity of the item, denoted by ξ_*i*_, and the speed latent variable:

(3) $$\begin{array}{r} f\left(t_{pi}|\tau_{p}\right)=\ln N\left(\xi_{i}-\tau_{p};\sigma_{i}^{2}\right) \end{array}$$

where $\sigma_{i}^{2}$ is the residual variance of the log-transformed response time. Here, $\frac{1}{\sigma_{i}^{2}}$ can be considered a time discrimination parameter since the smaller $\sigma_{i}^{2}$ is, the larger the proportion of the variance of response times explained by speed is. This model can also be seen as a constrained linear factor model with all factor loadings equal to each other (Molenaar et al., 2015b). Alternatively, one can use an unconstrained linear factor model with additional item-specific factor loadings (Fox et al., 2007). Different choices for the response time model, used as an ingredient for the hierarchical model, include a model based on Box-Cox transformation of response times (Klein Entink et al., 2009), and a Weibull model (Rouder et al., 2003).

For the relationship between the latent variables and for the relationship between the item parameters we use multivariate normal distributions. For identification, the mean vector of the latent variables is constrained to zero, and the variance of θ is constrained to one^1^. For the relationship between the item parameters (*α*_*i*_, *β*_*i*_, ξ_*i*_) we also use a multivariate normal distribution. Unlike the distribution of the person parameters, here the mean vector and the covariance matrix can be estimated freely.

The conditional independence assumption in Equation (1) means that accuracy and time can be correlated only if ability and speed, which determine their expected values, are correlated. The residual response accuracy and residual log-transformed response time are taken to be noise and the fluctuations on the response accuracy and response time sides of the model are taken to be uncorrelated.

## Modeling conditional dependence between time and accuracy

The conditional independence assumption can be relaxed and the relationship between residual response time and residual response accuracy can be incorporated into the model. One way to do that is to model the joint distribution of time and accuracy to the same item as a bivariate distribution with a non-zero correlation parameter. Ranger and Ortner (2012) proposed modeling the joint distribution of log-transformed response time (denoted by $t_{pi}^{*}$) and augmented continuous response accuracy (denoted by $x_{pi}^{*}$ defined such that $x_{pi}=I\left(x_{pi}^{*}>0\right)$) as a bivariate normal distribution with an item-specific conditional correlation, denoted by ρ_*i*_:

(4) $$f(x_{pi}^{*},t_{pi}^{*}|\theta_{p},\tau_{p})=N_{2}\left(\left[\begin{array}{c} \alpha_{i}\theta_{p}+\beta_{i}\\ \xi_{i}-\tau_{p} \end{array}\right],\left[\begin{array}{cc} 1 & \rho_{i}\sigma_{i}\\ \rho_{i}\sigma_{i} & \sigma_{i}^{2} \end{array}\right]\right).$$

Here, the marginal distribution of response accuracy and response time are the two-parameter normal-ogive model and log-normal model, the same as in the hierarchical model presented in the previous section. Meng et al. (2015) have further extended this model to allow the conditional correlation to vary, not only across persons, but also across items.

Bolsinova et al. (2017b) have shown that the joint model in Equation 4 is equivalent to a model in which the joint distribution of accuracy and time is factorized as a product of the marginal log-normal model for time and a conditional model for accuracy given time, which is a two-parameter normal-ogive model, with the intercept being a linear function of the standardized difference between the observed and expected log-transformed response time:

(5) $$\begin{array}{r} \mathrm{Pr}\left(X_{pi}=1|t_{pi},\theta_{p},\tau_{p}\right)=\text{Φ}\left(\alpha_{i}\theta_{p}+\beta_{i0}+\beta_{i1}\frac{\ln\;t_{pi}-\left(\xi_{i}-\tau_{p}\right)}{\sigma_{i}}\right), \end{array}$$

where *β*_*i*0_ is the baseline intercept and *β*_1*i*_ is the linear effect of standardized residual log-transformed response time on the intercept of the ICC. In addition to the linear effect on the intercept, the model can be extended with a linear effect on the slope of the ICC (Bolsinova et al., 2017b)^2^:

(6) $$\begin{array}{r} \mathrm{Pr}\left(X_{pi}=1|t_{pi},\theta_{p},\tau_{p}\right)=\text{Φ}\left(\left(\alpha_{i0}+\alpha_{i1}z_{pi}\right)\theta_{p}+\beta_{i0}+\beta_{i1}z_{pi}\right), \end{array}$$

where *z*_*pi*_ denotes the standardized difference between the observed and expected log-transformed response time $\frac{\ln\;t_{pi}-\left(\xi_{i}-\tau_{p}\right)}{\sigma_{i}}$, and *α*_*i*0_ and *α*_*i*1_ are the baseline slope and the linear effect of *z*_*pi*_ on the slope of the ICC, respectively. The parameters *β*_*i*1_ and *α*_*i*1_ can be interpreted as the main effect of residual log-transformed response time on response accuracy, and the interaction effect between ability and *z*_*pi*_ on accuracy, respectively. Throughout the paper we refer to this model as the linear conditional dependence model.

The approaches discussed above treat the response time as a continuous variable and relate the parameters of the IRT model for accuracy to deviations of the observed log-response time from its expected value. An alternative proposal has been to categorize response time into two classes—fast and slow—and jointly model the dichotomized response time and response accuracy using an IRTree model (De Boeck and Partchev, 2012). In this case, the ICC parameters can differ between the two classes (Partchev and De Boeck, 2012; DiTrapani et al., 2016). If the two-parameter normal-ogive model is used, then the probability of a correct response given response time is:

(7) $$\begin{array}{l} \mathrm{Pr}(X_{pi}=1|t_{pi},\theta_{p})=\Phi (\left(\alpha_{iF}ℐ\left(t_{pi}\leq {\tilde{t}}_{i}\right)+\alpha_{iS}\left(t_{pi}>{\tilde{t}}_{i}\right)\right)\theta_{p}\\ \;+\beta_{iF}ℐ\left(t_{i}\leq {\tilde{t}}_{i}\right)+\beta_{iS}ℐ\left(t_{i}>{\tilde{t}}_{i}\right)\text{​}), \end{array}$$

where ${\tilde{t}}_{i}$ denotes the median response time to item *i*, and subscripts *F* and *S* denote the fast and the slow class, respectively. Since, only two classes of response time are defined, only a monotonic relationship between response time and accuracy can be explored, for example responses in the slow class being more often correct than responses in the fast class (*β*_*iS*_ > *β*_*iF*_), or responses in the slow class being less informative about ability than responses in the fast class (*α*_*iS*_ < *α*_*iF*_).

It is important to note that separation of the response times into two classes is typically done using an item-level median split. Therefore, this approach is different from the linear models discussed above, since the ICC parameters are related to the categorized *observed* response time, and not to the difference between the expected and observed response time, such that the differences between the fast and slow classes capture not only the conditional dependence, but also the relationship between ability and speed (persons for whom the responses to item *i* are categorized as fast on average would have a higher speed latent variable in the log-normal model than persons for whom the responses to item *i* are slow).

## Modeling nonlinear conditional dependence

The linear conditional dependence models and the fast-slow model provide quite a simplistic picture of the relationship between response time and accuracy. The residual dependence between time and accuracy is not necessarily monotone and the change of the ICC parameters is not necessarily linear in *z*_*pi*_. To further investigate the relationship between response time and accuracy, we propose two new joint models for conditional dependence between response time and accuracy, and also use a nonparametric moderation method to explore the relationship between the residual log-transformed response time and the parameters of the response accuracy model.

### Joint model for quadratic conditional dependence

To allow for a nonlinear relationship between residual log-transformed response time and the ICC parameters, we extend the conditional model of response accuracy in Equation (6) with quadratic effects. To simplify the notation, we introduce a function Ψ(·, *x*) = (Φ(·))^*x*^(1−Φ(·))^1−*x*^. The resulting joint model for time and accuracy is then the following:

(8) $$\begin{array}{l} f(x_{pi},t_{pi}|\theta_{p},\tau_{p})=f(x_{pi}|t_{pi},\theta_{p},\tau_{p})f(t_{pi}|\tau_{p})\\ \;=\Psi ((\alpha_{i0}+\alpha_{i1}z_{pi}+\alpha_{i2}z_{pi}^{2})\theta_{p}+\beta_{i0}\\ \;+\beta_{i1}z_{pi}+\beta_{i2}z_{pi}^{2},x_{pi})\\ \;\frac{1}{t_{pi}\sqrt{2\pi }\sigma_{i}}\exp\left(-\frac{{\left(\ln t_{pi}-\xi_{i}+\tau_{p}\right)}^{2}}{2\sigma_{i}^{2}}\right), \end{array}$$

where *α*_*i*2_ and *β*_*i*2_ are the quadratic effects of the residual log-transformed response time on response accuracy. If *α*_2*i*_ < 0, then the strength of the relationship between ability and the probability of a correct response first increases with residual log-transformed response time and then decreases, and vice versa if *α*_*i*2_>0. Similar interpretations can be given to the sign of *β*_*i*2_. When the quadratic effect is negative, the corresponding parameter of the ICC (i.e., slope or intercept) is the highest when $z_{pi}=-\frac{\alpha_{i1}}{2\alpha_{i2}}$.

Our joint model is an extension of the hierarchical model, therefore in addition to the specification of the joint distribution of the outcome variables, we also need to specify the distribution of the latent variables and the distribution of the item parameters. On the person side we use $N_{2}\left(\text{0},\text{Σ}\right)$ where the variance of θ is contrained to be 1. On the item side, we use $N_{7}\left({\text{μ}}_{I},{\text{Σ}}_{I}\right)$ for {*α*_*i*0_, *α*_*i*1_, *α*_*i*2_, *β*_*i*0_, *β*_*i*1_, *β*_*i*2_, ξ_*i*_}, where ***μ***_*I*_ and **Σ**_*I*_ are the mean vector and the covariance matrix of the item parameters, respectively. Note, that while we are including nonlinear effects in modeling the conditional dependence between time and accuracy given ability and speed, we do not extend the standard hierarchical model with nonlinear effects on the higher level, since it goes beyond the scope of the current paper. However, one may consider more complex models for the joint distribution of the person parameters and for the joint distribution of the item parameters that would allow for a nonlinear relationship on the higher level as well as on the lower level.

This extended joint model for conditional dependence between response time and accuracy can be estimated in a similar way as the linear conditional dependence models (Bolsinova et al., 2017b) using Bayesian estimation. The Appendix contains the full specification of the density of the data, prior and posterior distributions, and the detailed steps of the Gibbs Sampler, in which the parameters are consecutively sampled from their full conditional posteriors.

### Multiple-category conditional dependence model

An alternative to the quadratic conditional dependence model for exploration of nonmonotone dependence is an extension of the slow-fast model. Allowing the ICC parameters to differ not just across two classes of responses, but across multiple classes, makes it possible to uncover nonmonotone relationships between residual response time and the ICC parameters (e.g., an item being most informative for the middle categories and least informative for the extreme categories).

Considering multiple categories is not the only way in which our joint model differs from the existing fast-slow models. Instead of categorizing the response time itself, we are going to use the residual log-transformed response time, since we are interested in the *conditional* dependence between response time and accuracy, taken separately from the relationship between speed and ability.

The joint distribution of response time and accuracy in this model is:

(9) $$\begin{array}{l} f(x_{pi},t_{pi}|\theta_{p},\tau_{p})=\Psi (\left(\alpha_{im}+\sum_{k=1,k\neq m}^{M}\alpha_{ik}z_{pik}^{*}\right)\theta_{p}\\ \;+\beta_{im}+\sum_{k=1,k\neq m}^{M}\beta_{ik}z_{pik}^{*},x_{pi})\\ \;\frac{1}{t_{pi}\sqrt{2\pi }\sigma_{i}}\exp\left(-\frac{{\left(\ln t_{pi}-\xi_{i}+\tau_{p}\right)}^{2}}{2\sigma_{i}^{2}}\right), \end{array}$$

where *M* is the number of categories of residual log-transformed response time, *m* is the baseline category, $z_{pik}^{*}=I\left(q_{k}\leq z_{pi}\leq q_{k+1}\right)$, and *q*_1_, …, *q*_*M*+1_ are the a priori defined thresholds between the categories (*q*_1_ = −∞, *q*_*M*+1_ = +∞). Note, that in this joint model response time is modeled as a continuous variable such that there is no loss of information in the measurement of speed due to categorization.

Given that residual log-transformed response time belongs to the baseline category, the item parameters are equal to {*α*_*im*_, *β*_*im*_}. When *z*_*pi*_ belongs to one of the remaining categories *k* ≠ *m*, the parameters are equal to {*α*_*im*_ + *α*_*ik*_, *β*_*im*_ + *β*_*ik*_}. When *M* > 2 the model allows for nonmonotone conditional dependence. For example, if *m* is the middle category and *β*_*ik*_ < 0, ∀*k* ≠ *m*, then it means that both responses that are slower than expected and those that are faster than expected are less often correct than responses for which the observed response time is closer to the expected response time. The more categories are used the more flexibly the model can account for different patterns of conditional dependence. However, the more categories there are the smaller the sample size per category is and the less precise the estimates of the item parameters are.

Analogous to the quadratic model, this joint model for time and accuracy can also be estimated using a Gibbs Sampler (see Appendix for details). Here we specify the same distribution for the latent variables, and similarly $N_{2M+1}\left({\text{μ}}_{I},{\text{Σ}}_{I}\right)$ is specified for {*α*_*i*1_, …, *α*_*iM*_, *β*_*i*1_, …, *β*_*iM*_, ξ_*i*_}.

### Nonparametric approach

The third approach to exploring nonlinear conditional dependence is in line with the nonparametric indicator-level moderation approach developed by Bolsinova and Molenaar (in press), which is a extension of the local structural equation modeling approach from Hildebrandt et al. (2016). The idea of the method is to explore the nonparametric relationship between the indicator-level covariate and the parameters of the latent variable model. In the case of investigating the conditional dependence between response time and accuracy, this method can be applied by using the residual log-transformed response time as the covariate for the intercept and the slope of the ICCs of the items in the accuracy measurement model. Using residual log-transformed response time instead of the observed response time itself is important because in that way one can investigate the relationship conditional on the latent variables and not the marginal relationship between time and accuracy. By including the residual log-transformed response time as a covariate in the analysis we can look at how the probability of a correct response changes depending on whether the response is shorter than expected or longer than expected (i.e., the intercept being a function of residual log-transformed response time) and how the relationship between ability and the probability of a correct response changes depending on the response being relatively fast or slow (i.e., the slope being a function of the residual log-transformed response time).

Unlike the first two approaches in which the joint distribution of response time and accuracy is modeled, in nonparametric moderation it is not possible to model the two outcome variables jointly since in this approach residual log-transformed response time is treated as an observed covariate. Therefore, we propose using a two-step procedure. First, the measurement model for response times is fitted and the estimates of the standardized residual log-transformed response time are computed:

(10) $$\begin{array}{r} {ẑ}_{pi}=\frac{\ln\;t_{pi}-{\hat{\xi }}_{i}+{\hat{\tau }}_{p}}{{\hat{\sigma }}_{i}}. \end{array}$$

Second, the estimates ẑ_*pi*_ are included in the analysis of response accuracy as indicator-level moderators.

For each item, a set of focal points *F*_1_, …, *F*_*J*_ for the value of the standardized residual log-transformed response time are defined for which the slope and intercept of the ICC are estimated. Since for all items the moderator has a mean of zero and a standard deviation of one, it makes sense to have the same focal points for different items. For each focal point *j* and for each item, the estimates of *α*_*ji*_ and *β*_*ji*_ are obtained by weighting the responses to the item from each person *p* using the distance between the value ẑ_*pi*_ and the focal point. For each combination of an item *i* and a focal point *j* a vector of weights **w**_*ji*_ is defined with each element corresponding to a particular person *p*:

(11) $$w_{pji}=\exp\left(-\frac{{\left({\hat{z}}_{pi}-F_{j}\right)}^{2}}{2{\left(hN^{-\frac{1}{5}}\right)}^{2}})\right),$$

there *h* is the bandwidth factor which serves as a smoothing parameter and determines how far from the focal point ẑ_*pi*_ has to be to have a relatively large impact on the estimates of the parameters *α*_*ji*_ and *β*_*ji*_. We will use the vale of 1.1 for *h*, which has been proposed in the nonparametric literature (Silverman, 1986) and has been successfully used for indicator-level moderation (Bolsinova and Molenaar, in press).

The item slopes and intercepts of the *K* items in the test are estimated in an iterative procedure. To start, the values of the slope and intercept are initialized for each combination of a person and an indicator, that is *N*×*K* matrices of response-specific slopes and intercepts, denoted by α^*^ and β^*^ respectively, are defined. The estimates of the item slopes and intercepts from the conditional independence hierarchical model can be used as starting values. After initialization, repeatedly for each item the estimates of *α*_*ji*_ and *β*_*ji*_ are obtained for each focal point *j* by maximizing the weighted log-likelihood:

(12) $$\begin{array}{l} \ln ℒ(\alpha_{ji},\beta_{ji}|X,\alpha^{*},\beta^{*},w_{ji})\\ \;=\sum_{p}\ln\int {\left(\Psi (\alpha_{ji}\theta +\beta_{ji},x_{pi})\right)}^{w_{pji}}\\ \;\prod_{k\neq i}\Psi (\alpha_{pk}^{*}\theta +\beta_{pk}^{*},x_{pk})N(\theta ;0,1)d\theta , \end{array}$$

where the responses to item *i* are weighted with **w**_*ji*_, while for the rest of the items *k* ≠ *i* the current values of response-specific slopes and intercepts contained in ${\text{α}}_{\cdot k}^{*}$ and ${\text{β}}_{\cdot k}^{*}$ are used.

After *α*_*ji*_ and *β*_*ji*_ are obtained, we update the values of ${\text{α}}_{\cdot i}^{*}$ and ${\text{β}}_{\cdot i}^{*}$ as follows:

(13) $$\alpha_{pi}^{*}=\{\begin{array}{ll} \alpha_{1i} & \text{ if }{\hat{z}}_{pi}<F_{1},\\ \alpha_{ji}+({\hat{z}}_{pi}-F_{j})\frac{\alpha_{(j+1)i}-\alpha_{ji}}{F_{j+1}-F_{j}} & \text{ if }F_{j}\leq {\hat{z}}_{pi}\leq F_{j+1},\forall j\in [1,J-1],\\ \alpha_{Ji} & \text{ if }{\hat{z}}_{pi}>F_{J}; \end{array}\%$$

with a similar specification for $\beta_{pi}^{*}$. That is, if ẑ_*pi*_ is outside of the range of the focal points, then the parameters are set equal to the parameters at the nearest focal point, and otherwise $\alpha_{pi}^{*}$ and $\beta_{pi}^{*}$ are computed using piece-wise linear regression.

Under this nonparametric approach the significance of conditional dependence can be tested using permutation tests. To perform these tests, one needs to repeatedly estimate the nonparametric relationship between the residual log-transformed response time and the parameters of the ICCs in permuted data sets, that is, data sets in which the response accuracy data points are kept intact but the residual log-transformed response times are randomly assigned to different persons in the sample. As a first tool to draw inferences about the significance of the relationship between the residual log-transformed response time and the ICC parameter, one can use graphical checks of deviations of the observed relationship and the relationship in the permuted data sets. However, a more rigorous test is to use the variance of the parameters across focal points as a statistic and compare the observed value to its distribution in the permuted data sets. The proportion of permuted data sets in which the variance is larger than in the observed data can be used to approximate the *p*-value for testing the hypothesis of conditional independence.

Furthermore, nonparametric moderation can be used to evaluate the viability of the assumption of linearity of conditional dependence. This can be done by performing posterior predictive checks (Meng, 1994; Gelman et al., 1996) for the linear dependence model. The idea of posterior predictive checks is to compare the observed relationship between the residual log-transformed response time and accuracy (as estimated using the nonparametric method) with its posterior predictive distribution under the linear conditional dependence model. To do so one needs to (1) sample from the posterior distribution of the model parameters of the linear conditional dependence model, (2) using the values of the parameters sampled from this posterior generate replicated data under the model, and (3) evaluate the relationship between residual log-transformed response time and the parameters of the ICCs in each of the replicated data sets using the nonparametric method. In addition to the visual comparison of the estimated relationship in the observed data set and multiple replicated data sets, one can also use some measure quantifying a deviation from linearity and compare the observed measure with its posterior predictive distribution in the replicated data sets. To obtain such a measure one can first compute residuals in a simple linear regression model with the estimates of the ICC parameter at focal points (${\hat{\alpha }}_{1i},\ldots ,{\hat{\alpha }}_{Ji}$ or ${\hat{\beta }}_{1i},\ldots ,{\hat{\beta }}_{Ji}$) as an outcome variable and the focal points as a predictor, and then compute the maximum of the absolute value of the cumulative sum of these residuals. The higher this value, the larger the deviation from linearity is. The proportion of replicated data sets in which the deviation from linearity is larger than in the observed data approximates the posterior predictive *p*-value. Small posterior predictive *p*-values (i.e., below 0.05) indicate that the deviation from linearity in the observed data is too large to conclude that the assumption of linearity of conditional dependence is viable.

## Empirical example

### Method

To illustrate how the nonlinear conditional dependence between response time and accuracy can be investigated, the proposed methods were applied to a data set of a high-stakes arithmetic test^3^. One of the test versions with 38 items answered by 4,632 persons was available for analysis. For this data set several models were fitted: (1) the conditional independence model, (2) the linear conditional dependence model, (3) the quadratic conditional dependence model, and (4) the multiple-category conditional dependence model. In Model 4 we considered 5 categories for residual log-transformed response time and the thresholds between the categories were set equal to -1.5, -0.5, 0.5, and 1.5 (i.e., the thresholds are symmetric around zero and each two neighboring thresholds are one standard deviation away from each other), the middle category (i.e., the category where the response times are the closest to their expected values) was used as a baseline.

The four models were fitted using Gibbs Samplers with 10,000 iterations including 5,000 iterations of burn-in. For the details of the estimation algorithm for the conditional independence model and the linear conditional dependence model see Bolsinova et al. (2017b). Gibbs Samplers for Models 3 and 4 are described in the Appendix. The fitted models were compared using the modified Bayesian information criterion (BIC) which has been previously used for comparing and selecting joint models for response time and accuracy (Bolsinova et al., 2017b)^4^. The criterion is modified in the sense that posterior means of the model parameters are used instead of the maximum likelihood estimates of the parameters. The models allowing for nonlinear conditional dependence have a larger penalty term based on their larger number of parameters (i.e., quadratic effects in addition to the baseline ICC parameters and the linear effects in the quadratic model, and category-specific ICC parameters for the multiple category model).

In addition to fitting the joint models for response time and accuracy, the nonparametric moderation method was applied to the data. To do so the standardized residuals of log-transformed response time in the one-factor model with equal factor loadings (i.e., which is equivalent to the log-normal model in Equation 3) were computed using “lavPredict” function from the R-package “lavaan” (Rosseel, 2012). As focal points we used [-2, -1.5, -1,-0.5, 0, 0.5, 1, 1.5, 2], that are the points where the observed log-transformed response time is equal to the expected value, and where the deviation from the expected value are equal to 0.5, 1, 1.5, and 2 residual standard deviations. To test the significance of the effect of residual log-response time on the slopes and the intercepts of the ICCs, permutation tests with 500 replications were performed.

Finally, to test the linearity of conditional dependence, posterior predictive checks were performed for the linear conditional dependence model. Given each 10th sample of the model parameters after the burn-in a replicated data set was generated under the linear conditional dependence model (i.e., 500 replicated data sets were generated). The nonparametric moderation method was applied for each of the replicated data sets in the same way as for the observed data. The relationship between standardized residual log-transformed response time and the ICC parameters in the replicated data sets and the observed data were compared graphically. Furthermore, in each data set for each effect, the maximum of the absolute value of the cumulative sum of the residuals in the simple linear regression model with the focal points as a predictor and the ICC parameter as an outcome variable was computed. For each effect, the proportion of replicated data sets in which the deviation from linearity (quantified by the maximum of the absolute value of the cumulative sum of the residuals) was larger than in the observed data was computed to approximate the posterior predictive *p*-value for the linearity check.

### Results

Table 1 shows the information criteria for the fitted joint models. The conditional independence model has the worst values compared to all models which take conditional dependence into account. This result shows that the conditional independence assumption does not hold for this test. Furthermore, models allowing conditional dependence to be nonlinear have lower information criteria values than the linear conditional dependence model, which shows that the assumption of linearity of conditional dependence also does not hold. The quadratic model was better than the multiple-category model, which points in the direction that the ICC parameters are not homogeneous within each category.

**Table 1:** Information criteria for the four joint models for time and accuracy.

**Model**	**-2LL**	***P***	**Modified BIC**
Conditional independence	2046864	–	2126317
Linear conditional dependence	2042274	76	2122368
Quadratic conditional dependence	2040146	152	2120882
Multiple-category model	2039572	304	2121591

*P is the number of additional parameters compared to the conditional independence model*.

It is important to investigate whether the main inferences that are made based on the linear conditional dependence model would also hold for the nonlinear conditional dependence models and for the nonparametric moderation method. The first question is about the presence of the effects on the intercept and the slope of the ICCs of the separate items. In the linear conditional dependence model for 24 and 30 items, the 95% credible intervals of *α*_1*i*_ and *β*_1*i*_ respectively, did not include zero, which can be seen as evidence of the presence of the effects. In the quadratic model for 33 and 37 items the 97.5% credible intervals^5^ of either *α*_*i*1_ or *α*_*i*2_, and of either *β*_*i*1_ or *β*_*i*2_ did not contain zero, which can be seen as evidence of the presence of conditional dependence for these items. In the multiple-category conditional dependence model for 29 and 37 items the 98.75% credible intervals^6^ of at least one of *α*_*ik*_, *k*≠*m* and one of *β*_*ik*_, *k*≠*m* did not contain zero. For 25 and 35 items the permutation test had *p*-values below 0.05 for the effects on the slopes and the intercept respectively, pointing to the presence of main and interaction effects of residual log-transformed response time on response accuracy.

**Figure 1:**
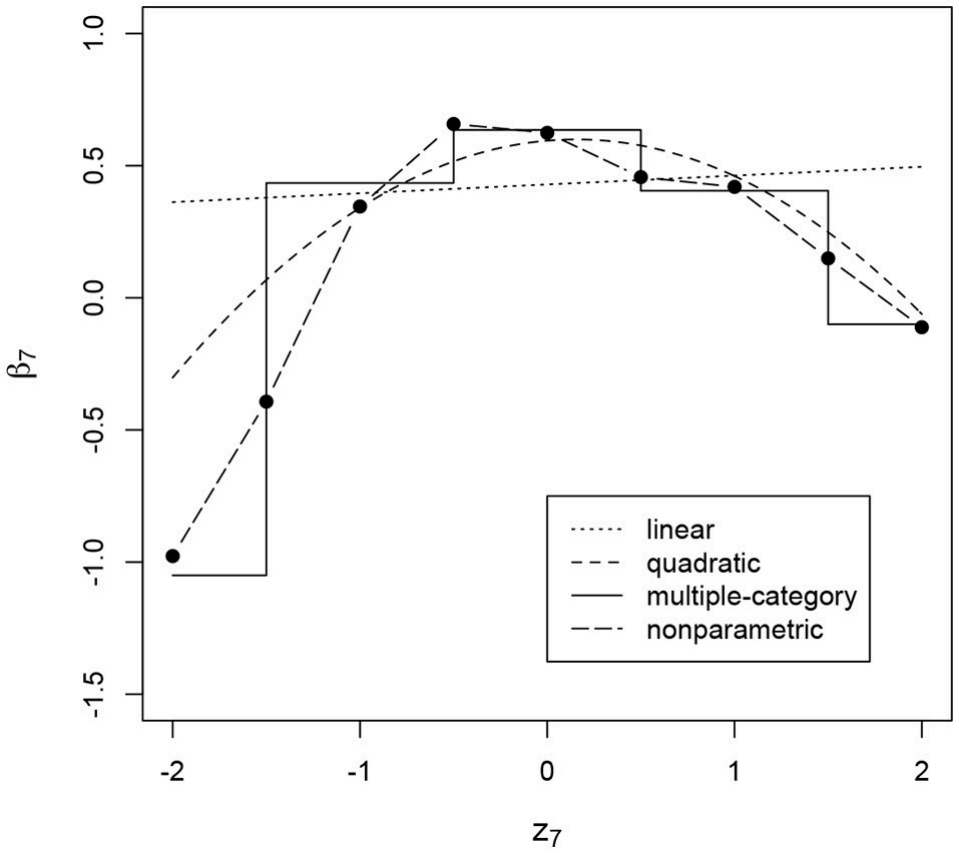
Intercept of the item characteristic curve of item 7 (*β*_7_, on the *y*-axis) as a function of residual log-transformed response time (*z*_7_, on the *x*-axis) estimated with different methods. The item intercept is modeled as a function of residual log-transformed response time, such that the intercept is different depending on the value of that residual.

We note that the nonlinear methods are more flexible and complex and therefore provide noisier results and have less power for detecting the effects, so it would not be surprising if a linear effect is detected by the simpler linear method, but not by more complex nonlinear methods. On the contrary, having items for which the linear conditional dependence model does not detect the effect, while it is detected by the nonlinear models should be worrying, since it would mean that the effect is not detected due to its nonlinear nature. This is the case, for example, for the effect on the intercept of item 7: Figure 1 shows the estimated relationship between the residual log-transformed response time and the intercept of the ICC for this item under the linear conditional dependence model and under the three nonlinear methods. It can be seen that when we allow the effect to be nonlinear and nonmonotone there is a clear relationship, while with the linear model the resulting relationship is close to a horizontal line.

**Figure 2:**
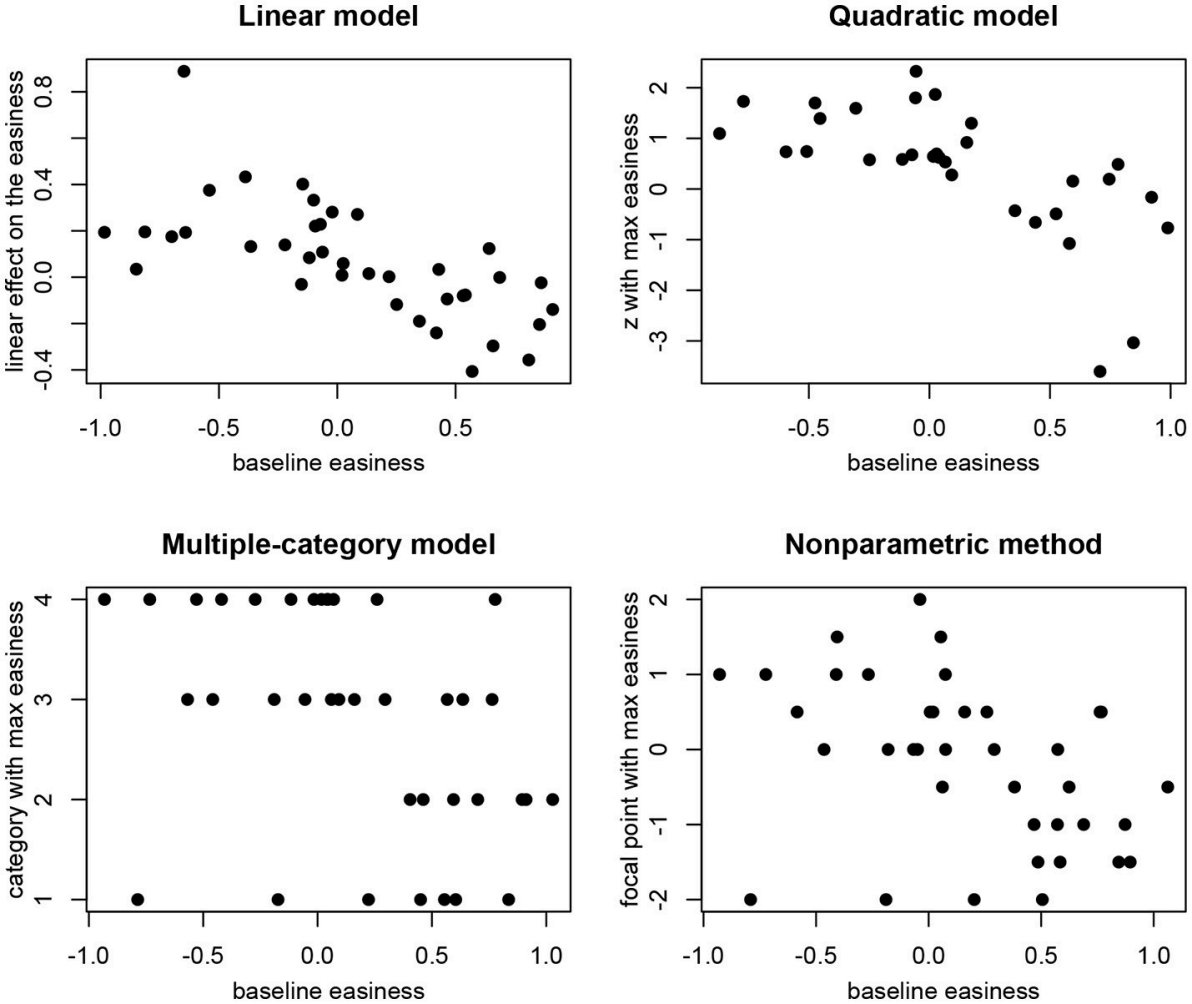
Differences in the effect of residual log-transformed response time (z) on item easiness depending on the baseline easiness.

The second kind of conclusion that is typically made based on the linear conditional dependence model is about the correlation between the baseline intercept of the items and the effect of residual log-transformed response time on the intercept. In multiple data sets previously this correlation was found to be negative (Bolsinova et al., 2017a,b). In our data set we found the same relationship. Figure 2 (top left) shows the relationship between the estimates of *β*_*i*0_ and *β*_*i*1_ in the linear conditional dependence model. For easier items the effects are more often negative, and for more difficult items the effects are more often positive. To check whether a similar conclusion would be made using the nonlinear methods we performed the following analyses: (1) For the quadratic model for the items with negative *β*_*i*2_ (i.e., items for which there exists a value of *z*_*pi*_ which maximizes the intercept of the ICC) we plotted the points at which the intercept is maximized ($-\frac{\beta_{i1}}{2\beta_{i2}}$) against the baseline intercept (see Figure 2, top right); (2) For the multiple-category model we plotted the category for which the item intercept is the highest against the intercept in the baseline category (see Figure 2, bottom left); (3) For the nonparametric method we plotted the focal points for which *β*_*ji*_ is the highest against the overall proportion of correct responses to the item (see Figure 2, bottom right). In all three additional plots we see a similar relationship as for the linear conditional dependence model: For easier items relatively fast responses tend to be most often correct, while for difficult items relatively slow responses tend to be most often correct.

The comparison of the information criteria shows that linearity of conditional dependence does not hold for the test as a whole. Additionally, we examined the estimates of the item hyper-parameters specifying the mean and the variance of the quadratic effects. The means of the quadratic effects across items were estimated to be -0.02 [-0.07, 0.04] for *α*_*i*2_s, and -0.09 [-0.15, -0.03] for *β*_*i*2_s. The variances of the quadratic effects were 0.03 [0.02, 0.05] for *α*_*i*2_s and 0.03 [0.02, 0.05] for *β*_*i*2_s. For the effects of the item intercepts there is a clear pattern of the intercept first increasing and then decreasing with residual log-transformed response time since the mean of *β*_*i*2_ is negative, but for the effects on the item slopes the pattern is not so clear.

**Figure 3:**
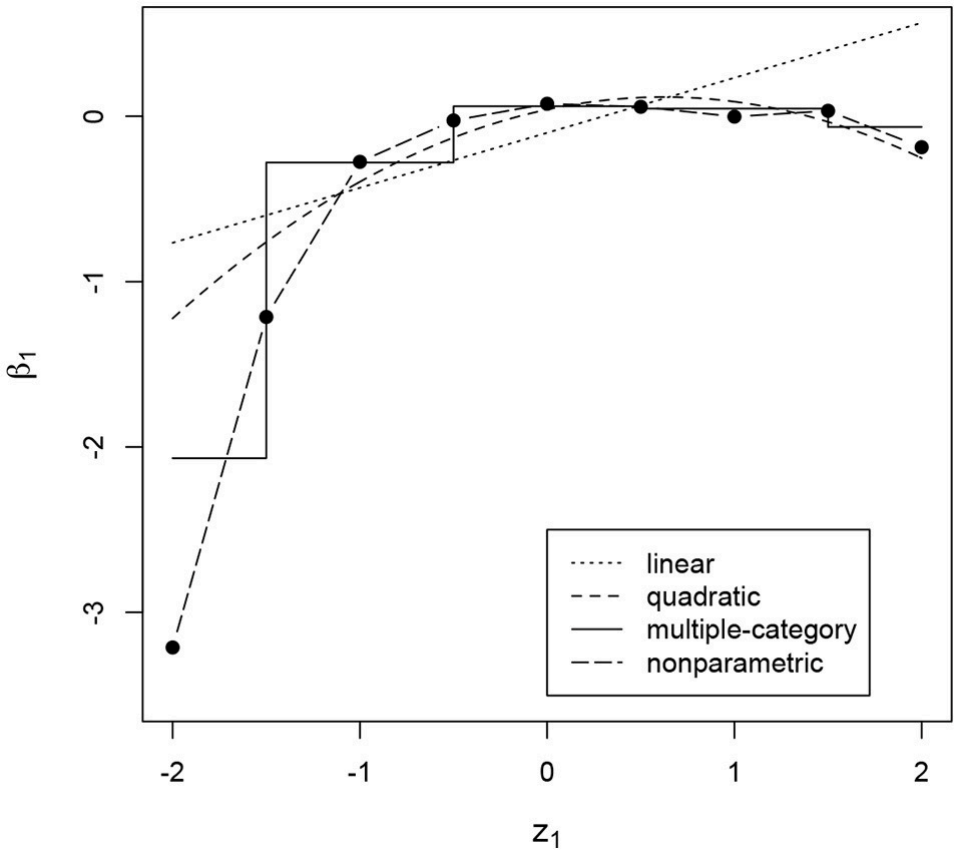
Intercept of the item characteristic curve of item 1 (*β*_1_, on the *y*-axis) as a function of residual log-transformed response time (*z*_1_, on the *x*-axis) estimated with different methods.

**Figure 4:**
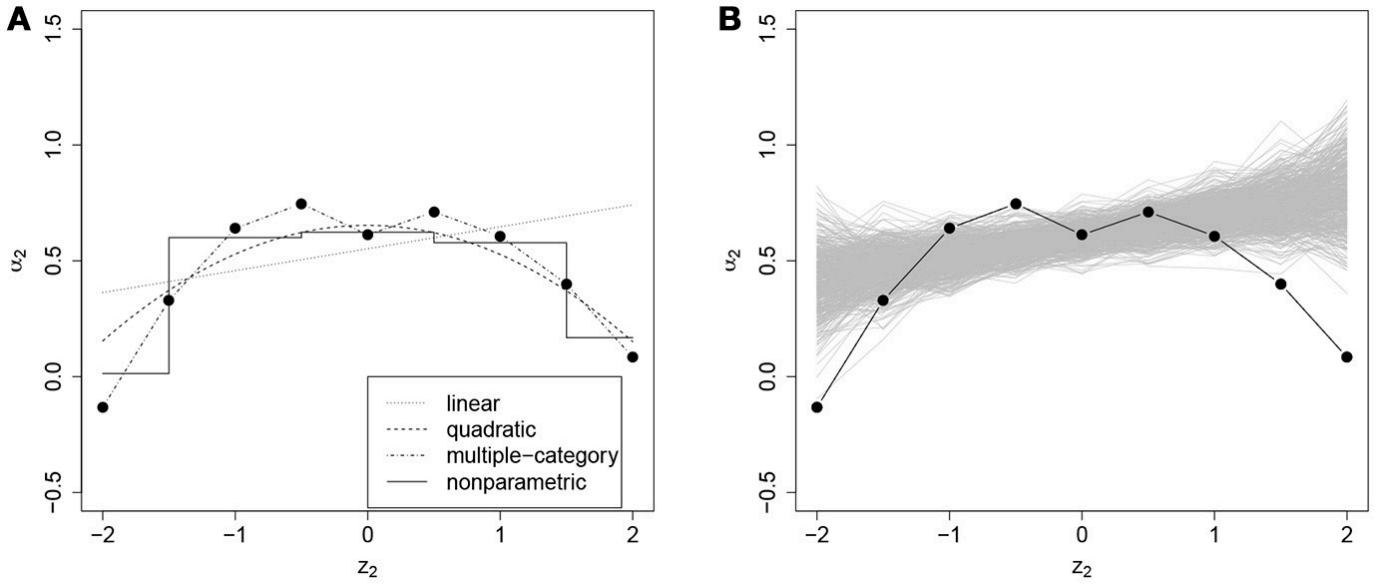
**(A)** Slope of the ICC of item 2 (*α*_2_, on the *y*-axis) as a function of residual log-transformed response time (*z*_2_, on the *x*-axis) estimated with different methods; **(B)** posterior predictive check for linearity of conditional dependence: each gray line represents the relationship between the residual log-transformed response time of item 2 (*z*_2_) and the slope of the ICC of item 2 (*α*_2_) estimated in the replicated data generated under the linear model, and the black line represents the relationship in the observed data.

**Figure 5:**
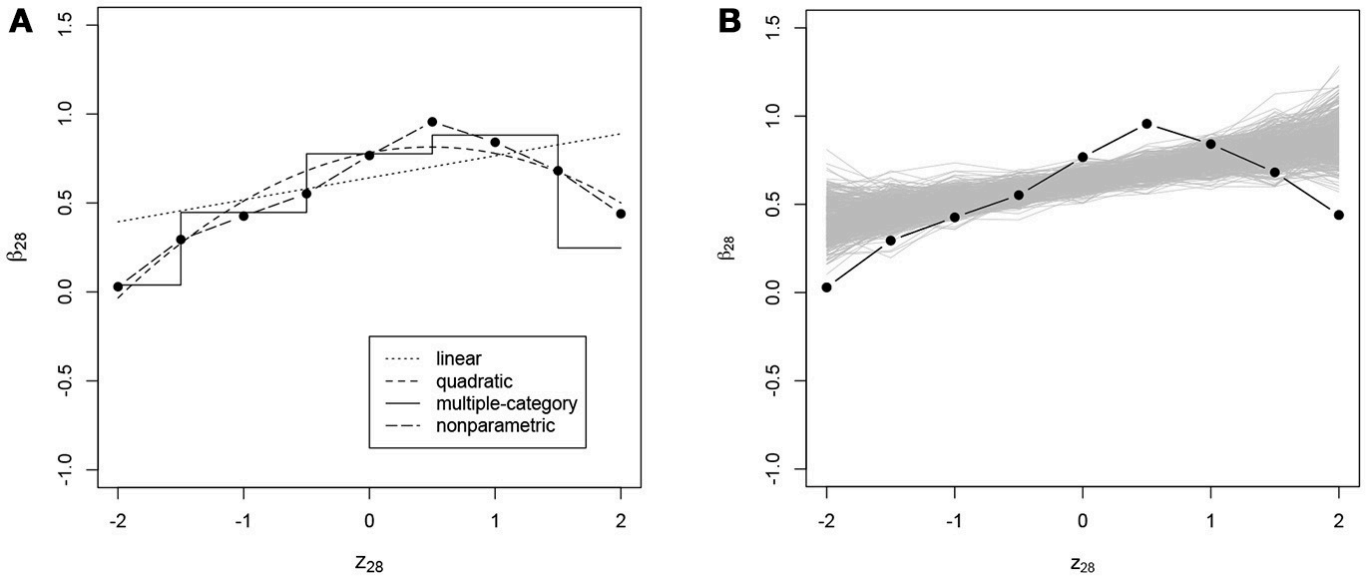
**(A)** Intercept of the ICC of item 28 (*β*_28_, on the *y*-axis) as a function of residual log-transformed response time (*z*_28_, on the *x*-axis) estimated with different methods; **(B)** posterior predictive check for linearity of conditional dependence: each gray line represents the relationship between the residual log-transformed response time of item 28 (*z*_28_) and the intercept of the ICC of item 28 (*β*_28_) estimated in the replicated data generated under the linear model, and the black line represents the relationship in the observed data.

**Figure 6:**
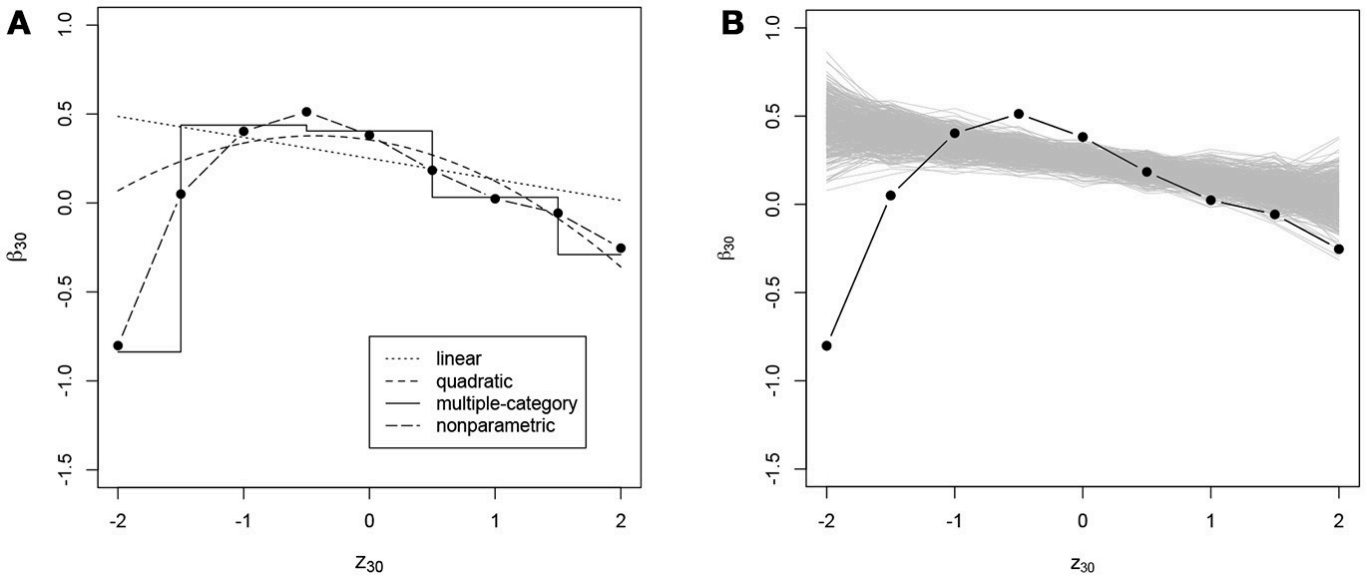
**(A)** Intercept of the ICC of item 30 (*β*_30_, on the *y*-axis) as a function of residual log-transformed response time (*z*_30_, on the *x*-axis) estimated with different methods; **(B)** posterior predictive check for linearity of conditional dependence: each gray line represents the relationship between the residual log-transformed response time of item 30 (*z*_30_) and the intercept of the ICC of item 30 (*β*_30_) estimated in the replicated data generated under the linear model, and the black line represents the relationship in the observed data.

In addition to the overall conclusions about the presence of nonlinear effects, at least for some of the items, it is also important to look at each item separately and evaluate the results of the posterior predictive checks for linearity. For 27 and 30 items the posterior predictive *p*-value for linearity was below 0.05 for the effects on the slope and the intercept of the ICC, respectively. Figures 3–6 give examples of some of the items with the largest deviations of conditional dependence from linearity. For item 1 the intercept of the ICC increases very steeply when the response is faster than expected, while positive deviations from the expected response time hardly result in further increase of the probability of a correct response (see Figure 3). From this figure, one can also see that the strength of the effect is underestimated in the linear conditional dependence model since the effect is averaged across the ranges of *z* in which there is an effect and where there is no effect. The slope of item 2 first increases and then decreases, for which the quadratic model gives quite a good approximation, while the linear conditional dependence severely misrepresents the relationship between residual log-transformed response time and the item slope (see Figure 4). For items 28 and 30 (see Figures 5, 6), the direction of the effect changes in the area where the observed response time is close to its expected value: Responses both faster than expected and slower than expected are less often correct than the responses with response times close to their expected values. Figures 5B, 6B illustrate the posterior predictive check for the intercepts of items 28 and 30. Here, the relationships in the observed data (black lines) clearly deviate from what would be expected if the data were generated under the linear conditional dependence model (gray lines). Note, that for the first of these two items the linear conditional dependence model reports a positive conditional dependence between response time and accuracy and for the second one it reports a negative conditional dependence, which is correct only for a part of the scale of the residual log-transformed response time and does not adequately represent the pattern of conditional dependence as a whole.

Additionally, we compared the estimates of ability under the conditional independence model, the linear conditional dependence model and the two nonlinear conditional dependence models (quadratic and multiple-category models) to check how the inclusion of conditional dependence in a model (and the exact way in which it is modeled) influences the inferences about the respondents. The correlations between the estimates of θ under each pair of models was very high, the lowest value of the correlation was above 0.988, and the highest value of the correlation was above 0.999. Therefore, in this example modeling conditional dependence does not change the measured construct, while it does allow learning more about the relationship between time and accuracy compared to the standard conditional independence model.

## Discussion

Our empirical example shows that conditional dependence between response time and accuracy can be nonlinear: in this example models allowing for nonlinear dependence are preferred over the linear dependence model, and for the majority of the items the posterior predictive checks indicate violations of linearity of the relationship between residual log-transformed response time and the ICC parameters. Using a linear conditional dependence model may in some situations lead to incorrect conclusions about the relationship between response time and accuracy: (1) One may conclude that conditional independence holds, when conditional independence is violated in a nonmonotone way such that the positive dependence in one range of the *z*-values and the negative dependence on another range cancel each other out; (2) The strength of the effect may be underestimated, when the effect is strong in some range of *z*-values and is either very weak or is absent in another range; (3) One may conclude that the dependence is, for example, negative while in fact it is both positive and negative depending on the range of *z*-values. In such situations, by modeling nonlinear conditional dependence one can get a better picture of the relationship between response time and accuracy in the data and get closer to understanding the response processes behind this relationship.

The approaches proposed in this paper make use of the difference between the observed and expected log-transformed response times, *z*_*pi*_, as a predictor variable to account for unobserved heterogeneity in the responses. In the model, we do not explicitly separate the unobserved heterogeneity by means of additional latent variables. As a result, *z*_*pi*_, which contains noise, is fully incorporated in the response model which decreases the power to detect an effect as the parameter estimates will have increased sampling fluctuations due to the noise in the residual log-transformed response time. However, we did not want to further complicate the model by introducing additional latent variables. In addition, introducing more latent variables may also decrease the power to detect an effect due to increased estimation error. Another aspect of the conditional dependence models is that false positives may arise if the response time model is misspecified. That is, such misspecifications will be absorbed in *z*_*pi*_ which in turn may be detected as a linear or non-linear conditional dependence effect if the misspecification is large enough. As a result, ideally one should carefully consider model fit of the response time measurement model before interpreting the results of the present parametric approach.

The conclusion about the negative relationship between the baseline intercept of the items and the effects of residual log-transformed response time on the intercept, previously found in other datasets (see e.g., Bolsinova et al., 2017b) and also found in our empirical example, seems to be robust regarding the violation of the linearity of the effect. With all three methods allowing for nonlinear dependence, we observed a relationship between the overall easiness of the item and the pattern of conditional dependence. When nonlinear conditional dependence is considered, we can no longer talk about the single effect on the intercept, instead we are considering the range of values of *z*_*pi*_ for which the intercept (and therefore response accuracy) is the highest. For easier items, the optimal values of *z*_*pi*_ tend to be more negative (responses faster than expected), while for difficult items, the optimal *z*_*pi*_ is positive (responses slower than expected).

In this paper we used three different approaches to modeling nonlinear conditional dependence: (1) the quadratic conditional dependence model, (2) the multiple-category conditional dependence model, and (3) the nonparametric modeling approach. These three approaches all have their comparative advantages and disadvantages. An important difference between the first two methods and the third one is that the first two methods allow modeling response time and accuracy jointly, while the third method requires a two-step procedure in which the estimates ẑ_*pi*_ are treated as observed covariates for the distribution of response accuracy. This can be seen as a disadvantage of the nonparametric approach. At the same time, the nonparametric approach allows for more flexibility in the relationship between residual log-transformed response time and the ICC parameters. A limitation of the quadratic approach is that it restricts the possible relationship between the residual log-transformed response time and the ICC parameters to having a particular parametric shape and does not allow exploration of the shape of the conditional dependence. One way in which the quadratic shape of the relationship between *z*_*pi*_ and the ICC parameters is restrictive is that the function is symmetric, whereas it could be that the decrease of the parameter when moving away from the maximum point (given that the quadratic effect is negative and there is a maximum) is stronger when *z*_*pi*_ is becomes smaller that its optimal value than when it becomes larger. The nonparametric method allows us to more closely follow the shape of the relationship, however due to its flexibility the method requires larger sample sizes. A limitation of the multiple-category approach is that it assumes that within each category of residual log-transformed response time the item parameters are constant, which might not necessarily be the case in practice.

While the empirical example considered an application from educational measurement, the developed methodology can be expected to be relevant for applications relating to ability measurement in general, in cases where both response time and accuracy are recorded. Like the traditional hierarchical model, the models proposed in this paper make it possible to obtain additional information about ability based on the observed response times, but the methods also allow one to further study and model the complex relationship that may exist between response time and accuracy. This can, for example, be considered relevant in the context of developing and applying intelligence tests or other complex cognitive tests, where one might expect that items display relevant patterns of conditional dependence. For example, it may be that response time is indicative of the particular problem solving strategy that a respondent employs, which may also affect how likely one is to provide a correct response. Additionally, it may be that long response times are indicative of aberrant test taking behavior, such as inattention or distraction, which makes it plausible that such responses should be seen as less informative of ability than responses for which the response times do not indicate aberrant behavior. Our methods allow one to take this into account, by allowing the discrimination parameter of the item to be influenced by residual response time. In this way, the proposed methods allow researchers to work with models for ability measurement that take both response time and accuracy into account and that are highly flexible with regard to the relationship between these two outcome variables that can be dealt with, and can accommodate a variety of deviations from conditional independence that can be expected in both high- and low-stakes psychological testing.

## Author contributions

MB and DM designed the study, MB wrote software, performed the analysis and wrote the paper, DM provided feedback on the manuscript.

### Conflict of interest statement

The authors declare that the research was conducted in the absence of any commercial or financial relationships that could be construed as a potential conflict of interest.

## Footnotes

^1^Note that if the factor model with item-specific factor loadings is used, then the variance of speed also has to be constrained.

^2^Note, that alternatively it has been proposed to include a linear effect on the log-transformed slope of the ICC (Bolsinova et al., 2017a)

^3^We would like to thank Dutch National Institute for Measurement in Education (CITO) for making this data set available to us. For confidentiality reasons we cannot disclose the content of the test items analyzed in this paper, but example items can be found at http://www.cito.nl/onderwijs/voortgezet%20onderwijs/rekentoets_vo/voorbeeldtoetsen. IRB approval was not needed for the study, since the data were collected previous to the study within high-stakes testing, and only response time and accuracy data and no information identifying the respondents was available for analysis.

^4^We are only using the modified BIC and not the modified Akaike information criterion (AIC) which has also been evaluated by the authors because they have shown that AIC tends to be too liberal.

^5^We decided to use a wider credible interval for the quadratic model because here two parameters are evaluated for each ICC parameter to make a conclusion about the presence of the effect instead of one, that is the area outside of the credible interval was divided by the number of parameters which were evaluated.

^6^We decided to use a wider credible interval for the multiple-category model because here four parameters are evaluated for each ICC parameter to make a conclusion about the presence of the effect instead of one parameter, that is the area outside of the credible interval was divided by the number of parameters which were evaluated.
