# Supplementary material for: Modeling Nonlinear Conditional Dependence Between Response Time and Accuracy
Source: Front Psychol. 2018 Sep 7;9:1525. doi: 10.3389/fpsyg.2018.01525 (PMC6137682; doi:10.3389/fpsyg.2018.01525)
Supplement: Supplementary file 1 [file Presentation_1.pdf]

Appendix

Estimation of the quadratic conditional dependence model and the multiple-category conditional dependence model

Estimation of the two proposed conditional dependence models can be done in a similar way. The joint posterior distribution of the model parameters is proportional to the product of the density of the data and the joint prior distribution

$$f(\boldsymbol{\alpha}, \boldsymbol{\beta}, \boldsymbol{\xi}, \boldsymbol{\sigma}^2, \boldsymbol{\theta}, \boldsymbol{\tau}, \boldsymbol{\mu}_I, \boldsymbol{\Sigma}_I, \boldsymbol{\Sigma}_P \,|\, \mathbf{x}, \mathbf{t}) \propto$$
$$\prod_p \prod_i \Psi\left(\boldsymbol{\alpha}_{i\cdot}\mathbf{v}_{pi}^T\theta_p + \boldsymbol{\beta}_{i\cdot}\mathbf{v}_{pi}^T, x_{pi}\right) \mathcal{N}(\ln t_{pi}; \xi_i - \tau_p, \sigma_i^2) \times$$
$$\prod_i \mathcal{N}_S([\boldsymbol{\alpha}_{i\cdot}, \boldsymbol{\beta}_{i\cdot}, \xi_i]^T; \boldsymbol{\mu}_I, \boldsymbol{\Sigma}_I) \mathcal{IG}(\sigma_i^2; 0.001, 0.001) \prod_p \mathcal{N}_2([\theta_p, \tau_p]^T; \mathbf{0}, \boldsymbol{\Sigma}_P)$$
$$\mathcal{N}_S(\boldsymbol{\mu}_I; \mathbf{0}, 100\mathbf{I}_S) \mathcal{IW}(\boldsymbol{\Sigma}_I; 2+S, \mathbf{I}_S) \mathcal{IW}(\boldsymbol{\Sigma}_P; 4, \mathbf{I}_2) \quad (1)$$

where $\boldsymbol{\theta}$ and $\boldsymbol{\tau}$ are vectors of length N (sample size); $\boldsymbol{\alpha}$ and $\boldsymbol{\beta}$ are $K \times 3$ matrices in the quadratic model and $K \times M$ matrices in the multiple-category model; $\boldsymbol{\mu}_I$ and $\boldsymbol{\Sigma}_I$ are a vector of length S and an $S \times S$ matrix, respectively, where S is equal to 7 in the case of the quadratic model and to $(2M+1)$ in the case of the multiple-category model; in the case of the quadratic model

$$\mathbf{v}_{pi} = \left[1, \frac{\ln t_{pi} - \xi_i - \tau_p}{\sigma_i}, \frac{(\ln t_{pi} - \xi_i - \tau_p)^2}{\sigma_i^2}\right]$$

and in the case of the multiple-category model

$$\mathbf{v}_{pi} = \left[1, \mathcal{I}\left(q_1 \leq \frac{\ln t_{pi} - \xi_i - \tau_p}{\sigma_i} \leq q_2\right), \ldots, \mathcal{I}\left(q_M \leq \frac{\ln t_{pi} - \xi_i - \tau_p}{\sigma_i} \leq q_{M+1}\right)\right],$$

For the residual variances of log-response time a vague inverse-gamma distribution is used. For the hypermean and hyper-covariance matrix the vague multivariate normal and vague inverse-Wishart distributions are used, respectively. For computational convenience, the hyper-covaraince matrix of the person parameters is freely estimated (with a corresponding vague inverse-Wishart distribution), but at each iteration all the model parameters are re-scaled such that the variance of θ is equal to 1.

The model can be estimated by obtaining samples from the posterior distribution using a Gibbs Sampler and using the posterior means and credible intervals of the parameters as their point estimates and measures of uncertainty, respectively. Data augmentation (Tanner & Wong, 1987) is implemented for simplifying the full conditional posteriors of the model parameters. For each response x_{pi} an augmented continuous variable $y_{pi} \sim \mathcal{N}(\boldsymbol{\alpha}_{i\cdot}\mathbf{v}_{pi}^T\theta_p + \boldsymbol{\beta}_{i\cdot}\mathbf{v}_{pi}, 1)$ is introduced (Albert, 1992), defined in such a way that $x_{pi} = \mathcal{I}(y_{pi} \geq 0)$. Hence, in the Gibbs Sampler samples are obtained from the following joint posterior distribution:

$$p(\mathbf{y}, \boldsymbol{\alpha}, \boldsymbol{\beta}, \boldsymbol{\theta}, \boldsymbol{\xi}, \boldsymbol{\sigma}^2, \boldsymbol{\tau}, \boldsymbol{\mu}_I, \boldsymbol{\Sigma}_I, \boldsymbol{\Sigma}_P \,|\, \mathbf{x}, \mathbf{t}). \tag{2}$$

In the Gibbs Sampler the model parameters are consecutively sampled from their full conditional posterior distributions which are specified in the steps below.

Step 1. For each combination of person p with item i sample the augmented response

y_{pi} from its full conditional posterior:

$$y_{pi} \sim \mathcal{N}(\boldsymbol{\alpha}_{i\cdot}\mathbf{v}_{pi}^T\theta_p + \boldsymbol{\beta}_{i\cdot}\mathbf{v}_{pi}^T, 1)\left(x_{pi}\mathcal{I}(y_{pi} \geq 0) + (1 - x_{pi})\mathcal{I}(y_{pi} < 0)\right), \tag{3}$$

which is a normal distribution truncated below zero if the response x_{pi} is correct, or above zero if the response is incorrect.

Step 2. For each person p sample the person parameters:

Step 2a. Sample the ability parameter θ_p from its full conditional posterior:

$$\theta_p \sim \mathcal{N}\left(\frac{\sum_i \boldsymbol{\alpha}_{i\cdot}\mathbf{v}_{pi}^T\left(y_{pi} - \boldsymbol{\beta}_{i\cdot}\mathbf{v}_{pi}^T\right) + \frac{\mu_{\theta_p}^*}{\sigma_\theta^{2*}}}{\sum_i(\boldsymbol{\alpha}_{i\cdot}\mathbf{v}_{pi}^T)^2 + \frac{1}{\sigma_\theta^{2*}}}, \frac{1}{\sum_i(\boldsymbol{\alpha}_{i\cdot}\mathbf{v}_{pi}^T)^2 + \frac{1}{\sigma_\theta^{2*}}}\right), \tag{4}$$

where $\mu_{\theta_p}^*$ and σ_θ^{2*} are the conditional mean and the conditional variance of ability given τ_p.

Step 2b. Sample τ_p from its full conditional posterior which is proportional to the product of three terms:

$$f(\mathbf{x}_{p\cdot} \mid \tau_p, \mathbf{t}_{p\cdot}, \boldsymbol{\alpha}, \boldsymbol{\beta}, \theta_p, \boldsymbol{\xi}, \boldsymbol{\sigma}^2) f(\mathbf{t}_{p\cdot} \mid \tau_p, \boldsymbol{\xi}, \boldsymbol{\sigma}^2) f(\tau_p \mid \theta_p, \boldsymbol{\Sigma}_P) \tag{5}$$

that is, the density of the accuracy data of person p, the density of the response time data of person p and the conditional prior of τ_p given the current value of θ_p. This posterior does not have a closed form, therefore we are using a Metropolis-Hastings algorithm to sample from this distribution. First, a candidate value τ_p^* is sampled from the following normal distribution:

$$\tau_p^* \sim \mathcal{N}\left(\frac{\sum_i \frac{(\ln t_{pi}-\xi_i)}{\sigma_i^2} + \frac{\mu^*_{\tau_p}}{\sigma_\tau^{2*}}}{\sum_i \frac{1}{\sigma_i^2} + \frac{1}{\sigma_\tau^{*2}}}, \frac{1}{\sum_i \frac{1}{\sigma_i^2} + \frac{1}{\sigma_\tau^{2*}}}\right), \tag{6}$$

where $\mu^*_{\tau_p}$ and σ_τ^{2*} are the conditional mean and variance of τ_p given the current value of θ_p. This proposal distribution is proportional to the product of the second and the third terms in the conditional posterior. Second, the candidate value is accepted with the following probability:

$$\Pr(\tau_p \to \tau_p^*) = \min\left(1, \frac{f(\mathbf{x}_{p\cdot} \mid \tau_p^*, \mathbf{t}_{p\cdot}, \boldsymbol{\alpha}, \boldsymbol{\beta}, \theta_p, \boldsymbol{\xi}, \boldsymbol{\sigma}^2)}{f(\mathbf{x}_{p\cdot} \mid \tau_p, \mathbf{t}_{p\cdot}, \boldsymbol{\alpha}, \boldsymbol{\beta}, \theta_p, \boldsymbol{\xi}, \boldsymbol{\sigma}^2)}\right). \tag{7}$$

Step 3. For each item i sample the item parameters:

Step 3a. Sample the parameters $[\boldsymbol{\alpha}_{i\cdot}, \boldsymbol{\beta}_{i\cdot}]^T$ from a multivariate normal distribution with posterior covariance matrix

$$\boldsymbol{\Omega}_i = \left(\mathbf{u}^T\mathbf{u} + (\boldsymbol{\Sigma}^*)^{-1}\right)^{-1}, \tag{8}$$

where

$$\mathbf{u} = \begin{bmatrix} \theta_1\mathbf{v}_{1i} & \mathbf{v}_{1i} \\ \vdots & \vdots \\ \theta_p\mathbf{v}_{pi} & \mathbf{v}_{pi} \\ \vdots & \vdots \\ \theta_N\mathbf{v}_{Ni} & \mathbf{v}_{Ni} \end{bmatrix},$$

and posterior mean vector

$$\boldsymbol{\zeta}_i = \boldsymbol{\Omega}_i \left(\mathbf{u}^T \mathbf{y}_{\cdot i} + (\boldsymbol{\Sigma}^*)^{-1} \boldsymbol{\mu}_i^* \right), \tag{9}$$

where $\boldsymbol{\Sigma}^*$ and $\boldsymbol{\mu}_i^*$ are the covariance matrix and the mean vector of the multivariate normal conditional prior distribution of $[\boldsymbol{\alpha}_{i\cdot}, \boldsymbol{\beta}_{i\cdot}]^T$ given the current value of ξ_i.

Step 3b. Sample ξ_i from its full conditional posterior which is proportional to the product of three terms:

$$f(\mathbf{x}_{\cdot i} \,|\, \xi_i, \mathbf{t}_{\cdot i}, \boldsymbol{\alpha}_{i\cdot}, \boldsymbol{\beta}_{i\cdot}, \boldsymbol{\theta}, \boldsymbol{\tau}, \sigma_i^2) f(\mathbf{t}_{\cdot i} \,|\, \xi_i, \sigma_i^2, \boldsymbol{\tau}) f(\xi_i \,|\, \boldsymbol{\alpha}_{i\cdot}, \boldsymbol{\beta}_{i\cdot}, \boldsymbol{\mu}_I, \boldsymbol{\Sigma}_I), \tag{10}$$

that is, the density of the accuracy data of item i, the density of the response time data of item i and the conditional prior of ξ_i given the values of the other item parameters. This posterior does not have a closed form, therefore we are using a Metropolis-Hastings algorithm to sample from this distribution. First, a candidate value ξ_i^* is sampled:

$$\xi_i^* \sim \mathcal{N} \left(\frac{\frac{1}{\sigma_i^2} \sum_p (\ln t_{pi} + \tau_p) + \frac{\mu_{\xi_i}^*}{\sigma_\xi^{2*}}}{\frac{N}{\sigma_i^2} + \frac{1}{\sigma_\xi^{2*}}}, \frac{1}{\frac{N}{\sigma_i^2} + \frac{1}{\sigma_\xi^{2*}}} \right), \tag{11}$$

where $\mu_{\xi_i}^*$ and σ_ξ^{2*} are the conditional mean and variance of ξ_i given the current values of the other item parameters. This proposal distribution is proportional to the product of the second and the third terms in the conditional posterior. Second,

the candidate value is accepted with the following probability:

$$\Pr(\xi_i \to \xi_i^*) = \min\left(1, \frac{f(\mathbf{x}_{\cdot i} \,|\, \xi_i^*, \mathbf{t}_{\cdot i}, \boldsymbol{\alpha}_{i\cdot}, \boldsymbol{\beta}_{i\cdot}, \boldsymbol{\theta}, \boldsymbol{\tau}, \sigma_i^2)}{f(\mathbf{x}_{\cdot i} \,|\, \xi_i, \mathbf{t}_{\cdot i}, \boldsymbol{\alpha}_{i\cdot}, \boldsymbol{\beta}_{i\cdot}, \boldsymbol{\theta}, \boldsymbol{\tau}, \sigma_i^2)}\right). \tag{12}$$

Step 3c. Sample σ_i^2 from its full conditional posterior which is proportional to the product of three terms:

$$f(\mathbf{x}_{\cdot i} \,|\, \sigma_i^2, \mathbf{t}_{\cdot i}, \boldsymbol{\alpha}_{i\cdot}, \boldsymbol{\beta}_{i\cdot}, \boldsymbol{\theta}, \boldsymbol{\tau}, \xi_i) f(\mathbf{t}_{\cdot i} \,|\, \sigma_i^2, \xi_i, \boldsymbol{\tau}) f(\sigma_i^2) \tag{13}$$

that is, the density of the accuracy data of item i, the density of the response time data of item i and the prior of σ_i^2. This posterior does not have a closed form solution, therefore we are using a Metropolis-Hastings algorithm to sample from this distribution. First, a candidate value σ_i^{2*} is sampled from

$$\sigma_i^{2*} \sim \mathcal{IG}\left(\frac{N}{2} + 0.001, \frac{\sum_p (\ln t_{pi} - \xi_i + \tau_p)^2}{2} + 0.001\right) \tag{14}$$

which is proportional to the product of the second and the third terms in the conditional posterior. Second, the candidate value is accepted with the following probability:

$$\Pr(\sigma_i \to \sigma_i^{2*}) = \min\left(1, \frac{f(\mathbf{x}_{\cdot i} \,|\, \sigma_i^*, \mathbf{t}_{\cdot i}, \boldsymbol{\alpha}_{i\cdot}, \boldsymbol{\beta}_{i\cdot}, \boldsymbol{\theta}, \boldsymbol{\tau}, \xi_i)}{f(\mathbf{x}_{\cdot i} \,|\, \sigma_i^2, \mathbf{t}_{\cdot i}, \boldsymbol{\alpha}_{i\cdot}, \boldsymbol{\beta}_{i\cdot}, \boldsymbol{\theta}, \boldsymbol{\tau}, \xi_i)}\right). \tag{15}$$

Note, that the conditional posterior distributions of the parameters of the response time model $(\boldsymbol{\tau}, \boldsymbol{\xi}, \boldsymbol{\sigma}^2)$ depend not only on the response time data but also on the response accuracy data. This follows directly from the joint posterior distribution of all model parameters, and is motivated by the fact that response times and

accuracies are modelled jointly and the two parts of the model are inherently connected to each other. Alternatively, one may consider a different sampling scheme in which the third term is dropped from the conditional posteriors of the response times parameters (see Equations 18, 23, and 26). However, in this case part of the information about the relationship between time and accuracy would not be taken into account. From simulations comparing the two sampling schemes, we observed that although the estimates of the parameters obtained with the two sampling schemes are very close to each other (correlations above .999), the estimates obtained through the sampling scheme based on the full posterior distribution (i.e., Equations 20, 25, and 28) correspond to a higher value of log-likelihood compared to the estimates obtained through the sampling scheme in which the response time parameters are sampled only given the response time data.

Step 4: Sample the covariance matrix of person parameters from

$$p(\mathbf{\Sigma}_P \mid \boldsymbol{\theta}, \boldsymbol{\tau}) \propto p(\boldsymbol{\theta}, \boldsymbol{\tau} \mid \mathbf{\Sigma}_P) p(\mathbf{\Sigma}_P). \tag{16}$$

which given the Inverse-Wishart prior is also a inverse-Wishart distribution (see for example, Hoff (2009)):

$$\mathbf{\Sigma}_P \sim \mathcal{IW}\left(4 + N, \mathbf{I}_2 + \sum_p (\theta_p, \tau_p)(\theta_p, \tau_p)^T\right). \tag{17}$$

Step 5: Re-scale model parameters to equate the variance of θ to 1:

$$\begin{aligned}
\boldsymbol{\theta} &\rightarrow \frac{1}{\sigma_\theta}\boldsymbol{\theta}, \\
\boldsymbol{\alpha} &\rightarrow \sigma_\theta\boldsymbol{\alpha}, \\
\boldsymbol{\mu}_\alpha &\rightarrow \sigma_\theta\boldsymbol{\mu}_\alpha \\
\boldsymbol{\Sigma}_P &\rightarrow \begin{bmatrix} 1 & \rho_{\theta\tau}\sigma_\tau \\ \rho_{\theta\tau}\sigma_\tau & \sigma_\tau^2 \end{bmatrix}
\end{aligned} \tag{18}$$

Step 6: Sample the covariance matrix of the item parameters from

$$p(\boldsymbol{\Sigma}_I \,|\, \boldsymbol{\alpha}, \boldsymbol{\beta}, \boldsymbol{\xi}, \boldsymbol{\mu}_I) \propto p(\boldsymbol{\alpha}, \boldsymbol{\beta}, \boldsymbol{\xi} \,|\, \boldsymbol{\Sigma}_I, \boldsymbol{\mu}_I) p(\boldsymbol{\Sigma}_I). \tag{19}$$

which given the Inverse-Wishart prior is known to be an inverse-Wishart distribution (see for example, Hoff (2009)):

$$\boldsymbol{\Sigma}_I \sim \mathcal{IW}\left(2 + S + K, \mathbf{I}_S + \sum_i ([\boldsymbol{\alpha}_{i\cdot}, \boldsymbol{\beta}_{i\cdot}, \xi_i]^T - \boldsymbol{\mu}_I)([\boldsymbol{\alpha}_{i\cdot}, \boldsymbol{\beta}_{i\cdot}, \xi_i] - \boldsymbol{\mu}_I^T)\right), \tag{20}$$

where K is the number of items.

Step 7: Sample the mean vector of the item parameters from

$$p(\boldsymbol{\mu}_I \,|\, \boldsymbol{\alpha}, \boldsymbol{\beta}, \boldsymbol{\xi}, \boldsymbol{\Sigma}_I) \propto p(\boldsymbol{\alpha}, \boldsymbol{\beta}, \boldsymbol{\xi} \,|\, \boldsymbol{\mu}_I, \boldsymbol{\Sigma}_I) p(\boldsymbol{\mu}_I). \tag{21}$$

With a multivariate normal prior for $\boldsymbol{\mu}_I$, this conditional posterior is also a multi-

variate normal with a mean vector equal to

$$\left((100\mathbf{I}_S)^{-1}+K\mathbf{\Sigma}_I^{-1}\right)^{-1}\left(\mathbf{\Sigma}_I^{-1}\boldsymbol{\gamma}\right) \tag{22}$$

where $\boldsymbol{\gamma}$ is a vector of column sums of the matrix $[\boldsymbol{\alpha}, \boldsymbol{\beta}, \boldsymbol{\xi}]$, and the covariance matrix equal to $\left((100\mathbf{I}_S)^{-1} + K\mathbf{\Sigma}_I^{-1}\right)^{-1}$.

References

Albert, J. (1992). Bayesian estimation of normal ogive item response curves using Gibbs sampling. *Journal of Educational Statistics*, *17*, 251-269. doi: 10.2307/1165149

Hoff, P. D. (2009). *A first course in Bayesian statistical methods.* New York: Spinger. doi: 10.1007/978-0-387-92407-6

Tanner, M., & Wong, W. (1987). The calculation of posterior distributions by data augmentation. *Journal of the American Statistical Association*, *82*, 528-540. doi: 10.2307/2289457
